# Experiences of emergency care practitioners on pre-hospital rapid sequence intubation in South Africa

**DOI:** 10.4102/hsag.v31i0.3281

**Published:** 2026-09-29

**Authors:** Tebatso J. Mathobela, Andrew R. Mosiane, Tshikani L. Khoza, Nombeko Mshunqane

**Affiliations:** 1 Department of Emergency Medical Care and Rescue, Faculty of Health Sciences, Durban University of Technology, Durban, South Africa; 2 Department of Physiotherapy, Faculty of Health Sciences, University of KwaZulu-Natal, Durban, South Africa

**Keywords:** emergency care practitioner, rapid sequence intubation, pre-hospital, patient safety, clinical governance, emergency medical services

## Abstract

**Background:**

Pre-hospital Rapid Sequence Intubation (RSI) is a complex, high-stakes and time-sensitive procedure performed in unpredictable resource-constrained environments. Successful execution of pre-hospital RSI demands coordinated teamwork, thorough preparation and clear communication.

**Aim:**

This qualitative study explored the experiences of Emergency Care Practitioners (ECPs) during pre-hospital RSI.

**Setting:**

The study was conducted across three South African provinces: Gauteng, KwaZulu-Natal and the Western Cape.

**Methods:**

The study adopted a descriptive qualitative design, using focus group discussions (FGDs) to understand ECPs’ experiences during prehospital RSI. Six FGDs were conducted online via Microsoft Teams. All discussions were audio-recorded, transcribed and analysed using qualitative data analysis software, NVivo 13 (2020 R1).

**Results:**

A total of 39 ECPs voluntarily participated in six FGDs, representing both public and private sectors. The following themes emerged; system-driven administrative demands and procedural complexity, resource and workforce constraints affecting RSI performance and communication-related challenges and information gaps affecting clinical decision-making.

**Conclusion:**

The ECPs voice concerns about challenges that require Emergency Medical Services (EMS) management to implement supportive measures, including improved access to resources and equipment during pre-hospital RSI. Additionally, they argue that practitioner proficiency promotes the effective execution of pre-hospital RSI.

**Contribution:**

The findings of this study and the insights gained can guide EMS leadership and policymakers in formulating targeted strategies that will ensure the availability of essential resources and strengthen operational support to enhance the safety and quality of care delivered to critically ill or injured patients requiring RSI.

## Introduction

Early access to intubation and ventilation allows for optimal oxygenation in traumatic brain injury (TBI), reduces the risks of secondary brain injury and may improve clinical outcomes of severely injured and critically ill patients. Pre-hospital rapid sequence intubation (RSI) is recognised as one of the most critical and high-risk advanced airway procedures undertaken in emergency medical care (EMC), particularly in unpredictable, time-sensitive and resource-constrained environments. Rapid sequence Intubation is regarded as a lifesaving procedure and a gold standard for advanced airway management, mainly because of the favourable conditions it creates to facilitate endotracheal intubation (ETI) (Okubo et al. 2017; Wallace & Maguire 2014).

Rapid sequence intubation involves the sequential administration of a potent induction agent such as ketamine or etomidate, followed immediately by a neuromuscular blocking agent, typically succinylcholine or rocuronium, in a rapid succession to facilitate endotracheal intubation in patients with actual or impending airway compromise (Stein et al. 2011). The procedure includes protection against aspiration of gastric contents, provides access to the airway for intubation and permits pharmacologic control of adverse responses to illness, injury and intubation (Brown et al. 2015; Kovacs & Sowers 2018). When performed effectively, RSI can significantly improve patient outcomes, particularly in cases of severe traumatic brain injury, airway obstruction or impending respiratory failure (Bernard et al. 2010).

In developing countries, RSI in the pre-hospital setting is associated with favourable outcomes, whereas there is a demonstrable association between delayed intubation and increased mortality (Pillay & Hardcastle 2017).

Although pre-hospital RSI is now acknowledged as an effective and essential procedure for securing the airway, it remains a high-risk, high-skill procedure that demands rapid decision-making, advanced technical proficiency and coordinated teamwork often under significant time pressure within resource constraints and limited conditions. Despite its potential to improve patient outcomes, a range of operational, logistical, clinical and organisational challenges may hinder its timely and effective execution and compromise both procedural success and patient safety (Crewdson et al. 2019).

Studies in the pre-hospital setting have highlighted the complexity and high risk associated with advanced airway procedures like RSI, emphasising the need for appropriate strategies, optimal patient management and ensuring robust systems-level support (Crewdson et al. 2019; Fevang et al. 2017; Jarvis et al. 2022). In the South African setting, RSI was incorporated into the scope of practice for degree-qualified emergency care practitioners (ECPs) by the Health Professions Council of South Africa (HPCSA 2009). Subsequent studies in South Africa show an ongoing gap in essential RSI medication availability and equipment and weaknesses in system infrastructure requirements and clinical governance that undermine procedural safety (Botha, Lourens & Stassen 2021).

Together, these findings suggest that ECPs frequently encounter challenges that can degrade RSI quality, including staffing limitations, variable access to medications and monitoring and fragmented quality-assurance structures (Botha et al. 2021; Gunning et al. 2013; Stein et al. 2011).

Consensus statements and position papers emphasise that safe pre-hospital RSI requires carefully selected and trained practitioners, standardised protocols, cognitive aids, pre-planned team roles, access to appropriate drugs and equipment (including rescue devices and capnography) and strong governance frameworks (audit, feedback and continuous quality improvement) (Botha et al. 2021; Olvera et al. 2018).

Where these system components are fragile or absent, the risk of hypoxia, hypotension, misplacement of Endo-Tracheal Tube (ETT) and other adverse events increases, particularly in an austere environment (Jarvis et al. 2022, 2024; Lockey et al. 2017).

Beyond technical proficiency, effective communication and human factors are pivotal in the RSI procedure.

In real-world practice, pre-hospital emergency care providers often work under challenging conditions, including noisy environments and emotionally charged and hazardous situations that are inherent to the pre-hospital environment. Limited or absent access to a patient’s past or current medical history further constrains decision-making and drug selection, amplifying cognitive load and the potential for error. These non-technical stressors, such as miscommunication, role ambiguity and information gaps, are well-recognised contributors to task and cognitive overload during pre-hospital airway management (Beecham & Kohn 2025).

Section 47 of the *National Health Act (Act No. 61 of 2003)* requires all health establishments to meet specific quality standards relating to the provision of healthcare services. These standards are further put into practice through the Department of Health Regulations for Emergency Medical Services (EMS), which set out requirements for human resources, equipment, operational systems and procedures, patient safety and the delivery of emergency care services (National Department of Health 2015; South Africa, Department of Health 2003). In line with these requirements, RSI was formally incorporated into the scope of practice of pre-hospital ECPs in 2009 by the HPCSA, with the aim of improving patient outcomes in the pre-hospital setting. Despite its potential benefits, ECPs continue to face significant challenges in performing RSI effectively in the pre-hospital environment (Botha et al. 2021).

These challenges are frequently linked to inadequate resources and limited availability of essential equipment and medications, among others. These factors can disrupt procedural flow, delay critical interventions and increase the likelihood of technical errors, ultimately heightening the risk of adverse patient outcomes.

Although the HPCSA and existing literature emphasise the importance of robust clinical governance, quality assurance and adequate resourcing to ensure safe and effective RSI practice, there remains limited evidence on the magnitude and nature of these challenges within the South African pre-hospital context.

Given the complexities associated with pre-hospital RSI, this qualitative study aimed to explore and describe experiences of ECPs performing pre-hospital RSI within the South African setting. By gaining insights into these experiences, the study sought to identify challenges that influence RSI practice and generate evidence that may inform clinical governance, strengthen practitioner support systems and enhance the safety and quality of pre-hospital emergency care in South Africa.

## Research methods and design

A descriptive qualitative approach was adopted to explore the experiences of ECPs during pre-hospital RSI in South Africa. Focus group discussions were conducted to collect data and analysed using inductive Thematic analysis as guided by Braun and Clarke. As a result of the paucity of research exploring this phenomenon, a qualitative descriptive approach was deemed appropriate for this study.

### Study setting and population

The study was conducted across three South African provinces: Gauteng, KwaZulu-Natal and the Western Cape. These provinces were selected because they have the highest concentration of ECPs in South Africa.

The South African EMS system comprises both public and private sector providers, each operating within distinct organisational and operational frameworks. Public EMS systems are typically characterised by higher patient volumes, broader service coverage and resource constraints, whereas private EMS providers often have lower patient volumes, greater access to advanced equipment, structured clinical governance systems and additional operational resources. These contextual differences are known to influence clinical exposure, decision-making processes and the delivery of pre-hospital RSI.

The study population comprised operational ECPs employed within both public and private EMS sectors across the selected provinces. Participants were purposively recruited based on their experiences and involvement in pre-hospital RSI. Inclusion criteria required participants to be registered with the HPCSA, employed full-time in either operational or academic roles and actively involved in pre-hospital RSI.

Eligibility was confirmed during recruitment, where participants were asked to indicate their RSI experience in response to the study invitation. Only those with prior RSI experience were included. Individuals who did not meet these criteria were excluded. Inclusion of both public and private EMS participants positioned the study to explore potential similarities and differences in RSI practice and challenges across these two predominant operational settings in South African EMS.

A total of 39 ECPs participated in six FGDs. Participants were further grouped according to their work setting (public vs private EMS) to account for differences in operational context and clinical practice environments.

All participants from private EMS providers were grouped together, while those from public EMS providers were allocated to separate groups. The grouping strategy ensured that discussions reflected shared operational realities and facilitated a more meaningful exploration of sector-specific practices, challenges and experience related to pre-hospital RSI. Variations in group sizes were influenced by the availability of participants within each sector.

### Sampling and recruitment

A purposive sampling strategy was employed to recruit ECPs with relevant experience in pre-hospital RSI.

This approach ensured that participants possessed the necessary clinical expertise to meaningfully contribute to the study objectives. In addition, a snowball sampling technique was utilised to enhance recruitment by allowing participants to refer other eligible colleagues within their professional networks.

Participant recruitment was carried out in accordance with the ethical principles of autonomy, beneficence and justice. Access to participants was facilitated through EMS organisational management, who acted as gatekeepers. Gatekeepers were provided with the study information and participant recruitment letter via email, which they disseminated to eligible ECPs within their respective organisations. To maintain confidentiality, gatekeepers did not share any personal information of potential participants with the researcher.

Interested participants were required to initiate contact directly with the researcher via email or professional WhatsApp™ communication. This approach ensured voluntary participation and preserved participation autonomy. Furthermore, participants who received the study information were encouraged to share their invitation with other eligible colleagues who could independently contact the researcher if interested in participating.

All participants received an electronic information sheet and informed consent form prior to participation. Written informed consent was obtained electronically before data collection commenced. Participation was entirely voluntary, and participants were informed of their right to withdraw from the study at any stage without any consequences.

### Data collection

Six FGDs were conducted virtually via Microsoft Teams from 09 June 2025 to 26 June 2025, with each session lasting 60 min – 90 min. This online approach was used to facilitate participation from geographically dispersed participants and to address logistical challenges related to coordinating in-person discussions. To minimise potential researcher bias, the FGDs were chaired and facilitated by a trained research assistant, while an independent observer with experience in qualitative research attended to ensure consistency and quality of data collection. All discussions were audio-recorded through Microsoft Teams and subsequently transcribed verbatim using the platform’s transcription function. The primary researcher reviewed and refined the transcripts for accuracy before sending them to the participants for member checking. The transcripts were also reviewed by the research observer for additional verification.

### Data analysis

Inductive thematic analysis was undertaken following Braun and Clarke’s framework to systematically identify and interpret patterns within the transcript data. All transcripts were imported into NVivo13 (2020 Release 1 [Lumivero]) qualitative data analysis software for coding and organisation. The researcher generated preliminary codes, which were clustered into sub-themes and themes. These were refined and supported by participant’s quotations. Coding was conducted independently by the researcher and reviewed by co-authors. The discrepancies were resolved by consensus among the researcher and co-authors.

### Trustworthiness and bias

To enhance the trustworthiness of the study, Guba and Lincoln’s criteria of credibility, transferability, dependability and confirmability were applied. Credibility was strengthened through member checking and co-coding by the research team. Multiple FGDs conducted over a 3-week period allowed for data collection until saturation. Transferability was enhanced by providing rich details about the study setting, including the pre-hospital emergency care environment, participant characteristics (ECPs) and the use of online platforms for data collection. Dependability was ensured by maintaining detailed records, or a comprehensive audit trail, of every stage of the research, from recruiting participants and collecting data on Microsoft Teams to transcribing, coding and developing themes. Confirmability was achieved through self-reflection, transparent records and an audit trail. Having an independent research assistant and a research observer involved in data collection helped reduce researcher bias.

### Ethical consideration

Ethical clearance for the study was obtained from the Durban University of Technology Institutional Research Ethics Committee (ethics number: IREC 292/24). Additional approval to conduct the study was sought from the Gauteng Provincial Government Research Ethics Committee, KwaZulu-Natal Department of Health Research Committee, as well as the ethics committees of Netcare, ER24 and Rocket Helicopter Emergency Medical Services (HEMS).

The researcher upheld key ethical principles throughout the study, with particular emphasis on maintaining participant privacy and confidentiality. Focus group discussions were conducted virtually via Microsoft Teams, with access restricted exclusively to enrolled participants. To protect anonymity, participants’ names were not used; instead, unique codes were assigned to all data. Digital files were secured with password encryption, and no identifiable information was linked to the research records. Informed consent was obtained voluntarily, with participants granting permission for audio recording without any form of coercion.

## Results

### An overview of themes

Three key themes emerged as challenges related to prehospital RSI: (1) System-driven administrative demands and procedural complexity, (2) resource and workforce constraints affecting RSI performance and (3) communication-related challenges and information gaps affecting clinical decision-making, as shown in Table 1.

**TABLE 1:** Themes and sub-themes.

Themes	Sub-themes
1. System-driven administrative demands and procedural complexity	1.1 Mandatory clinical consultation requirements 1.2 Extensive planning and preparation demands
2. Resources and workforce constraints impacting RSI performance	2.1 Reliance on unskilled or inexperienced assistants 2.2 Limited availability of essential equipment and medications 2.3 Limited personnel support during RSI procedures
3. Communication-related challenges and information gaps affecting clinical decision making	3.1 Limited access to accurate patient medical history 3.2 Miscommunication among healthcare providers

RSI, rapid sequence intubation.

#### Theme 1: System-driven administrative demands and procedural complexity

System-driven administrative demands and procedural complexity in this study refer to the regulatory, governance and procedural requirements imposed by emergency service providers as part of clinical governance intended to guide practitioners in delivering safe and effective patient care. These requirements often consume valuable time and resources, diverting practitioner attention away from direct clinical decision-making and potentially resulting in significant delays in patient management. In this study, participants identified mandatory clinical consultation prior to performing RSI as a key system-driven requirement. This process was reported to frequently cause significant delays, undermining preparation and planning for the procedure and ultimately increasing the risk of adverse events.

**Sub-theme 1.1: Mandatory clinical consultation requirements:** Participants reported that the mandatory requirement to contact an on-call doctor for permission to perform RSI, particularly in private emergency services, frequently leads to significant delays.

Participants expressed frustration as considerable time is spent on the phone during these consultations instead of performing the procedure. Participants reported that consultations not only hinder time-sensitive intervention but also make them appear unprofessional in front of patients and their families. This is reflected in the following quotes:

‘What I encountered from my part is I prepped everything, had drugs ready, but I was stuck on the phone consulting. If I recall correctly, when I hanged up the phone it was 36 minutes of consultation, and that was beyond even the preparation time for me.’ (FGD 4, P23, Private EMS)

‘So, second RSI was also a matter of a case of having to consult as well in a private company … which tend to cause some delays and also interferes or either over clouds, your judgement as well as a clinician.’ (FGD 4, P24, Private EMS)

‘Private service has so many protocols to go through before you actually RSI the patient, the one time it took like 20 minutes to just get hold of someone to actually approve.’ (FGD 4, P25, Private EMS)

‘Just trying to get hold of someone to get permission to RSI is really annoying.’ (FGD 4, P25, Private EMS)

**Sub-theme 1.2: Extensive planning and preparation demands:** Participants highlighted that inadequate time and failure to plan and prepare adequately for RSI often make the procedure challenging, particularly when faced with a difficult airway. This is reflected in the following quotes:

‘… that was from lack of preparation prior to initiating the process, and then one hypoxic event during intubation prehospitally again from lack of proper preparation.’ (FGD 6, P37, Public EMS)

‘… just proper planning and doing all your checks correctly will prevent a lot of things from happening, especially these adverse events.’ (FGD 5, P32, Public EMS)

‘So, it’s one of those procedures that you want to stick to … a checklist, particularly the RSI checklist … irrespective of environment, irrespective how of apparent the tube is required. It’s basically to get it right.’ (FGD 6, P34, Public EMS)

#### Theme 2: Resource and workforce constraints impacting rapid sequence intubation performance

Resource and workforce constraints impacting RSI performance in this study refer to situations where the demands of a procedure exceed the available human, technical or material resources, placing excessive strain on practitioners and potentially compromising the quality and safety of care. In this study, most participants indicated that RSI performance was often hindered by such constraints, primarily because of limited on-scene personnel, the presence of unskilled assistants and persistent shortages of functional medical equipment and essential medications.

**Sub-theme 2.1: Reliance on unskilled or inexperienced assistants:** Participants highlighted that a lack of access to knowledgeable assistants affected their proficiency in performing RSI. Many reported that their assistants were inexperienced and unfamiliar with the RSI process, leading them to perform the entire procedure alone, which significantly increases the difficulty of performing RSI effectively. This is reflected in the following quotes:

‘So now the problem is … I had enough hands for the RSI to go smoothly. But I ended up doing things alone because the people I was with, most of them didn’t understand what was going on.’ (FGD 1, P4, Public EMS)

‘I tend to find myself having to do a crash course with the crews around me because RSI is a very difficult thing to do on your own … so I find myself having to give instructions on the go and it becomes difficult.’ (FGD 3, P13, Private EMS)

‘There were also crews from the government sector … didn’t even know how to use BVM to say the least.’ (FGD 4, P19, Private EMS)

‘Sometimes you are on scene where you are the only one who actually knows what’s needs to be done. you need to prepare and explain the procedure to everyone else, but not everyone will understand it to your level.’ (FGD 5, P27, Public EMS)

‘I do agree with my colleagues where they say that basically as an ECP you’re very much alone when it comes to the RSI, unless you’re working by the ALS that knows the procedure.’ (FGD 5, P30, Public EMS)

**Sub-theme 2.2: Limited availability of essential equipment and medications:** A shortage of essential medical equipment and medications required for the effective execution of pre-hospital RSI was reported as hindering the ability of participants to perform RSI safely and efficiently, increasing the risk of potential adverse events. In some cases, the needed medical equipment was present but not functioning properly. This is reflected in the following quotes:

‘So, it was one of those scenarios where I lacked access to things like blood gas. So, due to lack of resources. The patient ended up crashing.’ (FGD 1, P5, Public EMS)

‘The problem is that sometimes I face a shortage of RSI drugs. Like right now, I’m only using Rocuronium, but my preferred drug of choice is succinylcholine because it’s short acting.’ (FGD 2, P12, Public EMS)

‘I had a very difficult intubation, and another thing is the alternative rescue devices like LMA, sometimes you have sometimes even if you have it’s not the right size. So basically, we are short of RSI drugs, and you only have one choice of drug.’ (FGD 2, P12, Public EMS)

‘My first attempt wasn’t good, and I was like, no, guys let’s pre-oxygenate again and then try again. But because I thought the BVM was working it wasn’t working.’ (FGD 5, P27, Public EMS)

**Sub-theme 2.3: Limited personnel support during rapid sequence intubation procedure:** Participants highlighted that, at times, they find themselves alone on the scene without any support from other pre-hospital emergency care personnel, making the execution of the RSI procedure extremely challenging. In some instances, they are compelled to rely on bystanders, who often lack any medical knowledge, to assist with the procedure. This is reflected in the following quotes:

‘… as soon as you find the patient, you realise the patient needs this procedure and you are alone. There’re maybe one or two bystanders. They have no clinical knowledge or whatsoever.’ (FGD 1, P4, Public EMS)

‘I was alone with for about 20 minutes until we waited for another service to arrive. But it just took really long … like we were on that scene for over an hour just because we had no backup.’ (FGD 4, P25, Private EMS)

‘… you alone on the scene and you have to make use of police and law enforcement officials to assist you in those cases.’ (FGD 6, P36, Public EMS)

#### Theme 3: Communication-related challenges and information gaps affecting clinical decision making

Communication-related challenges and information gaps affecting clinical decision-making in pre-hospital emergency care refer to obstacles that limit the effective exchange and availability of critical information between practitioners, patients and other healthcare providers. Participants in this study highlighted that one of the major challenges during RSI was the frequent lack of access to a patient’s past and current medical history, which hindered informed and timely clinical decision-making. In addition, miscommunication among practitioners and across healthcare providers was reported to further complicate the procedure, potentially increasing the likelihood of errors and adverse events.

**Sub-theme 3.1: Limited access to accurate patient medical history:** Limited access to accurate patient medical history frequently makes it challenging to understand the patient’s condition, which complicates the delivery of appropriate care. This is reflected in the following quotes:

‘It was an unknown history, but what I know is that the patient was hyperventilating, blood pressures were on the floor [slang: blood pressure was low], very low.’ (FGD 1, P5, Public EMS)

‘This was a bit of a difficult case for me because we didn’t really know what happened to the patient. They just found a patient on the side of the road in a polytrauma state. Nobody knows what happened.’ (FGD 3, P17, Private EMS)

‘While we we’re doing the vortex and then we found out later that the patient was heavily on substances which they had an influence on all the drugs we were using. Sometimes that information is not always available to us.’ (FGD 6, P35, Public EMS)

**Sub-theme 3.2: Miscommunications among healthcare providers:** Miscommunication among healthcare providers is a significant barrier to effective pre-hospital RSI. In some cases, unclear or incomplete information exchange leads to confusion, which can increase the risk of adverse events. This is reflected in the following quotes:

‘I feel like miscommunication between us and maybe the crews that you might be performing the RSI with also leads to many adverse events.’ (FGD 5, P32, Public EMS)

‘The ECP gave out an instruction that OK draw so much ketamine and give it to the patient. She didn’t specify the actual dose … the personnel took the whole ampoule of Ketamine, gave the whole drug … That was quite a lot of ketamine that pushed the patient into cardiac arrest.’ (FGD 5, P32, Public EMS)

‘I suspected one other clinician gave something ‘cause I remember they were drawing medication, When I turned around to the patient was in cardiac arrest … our communication also was not on par …’ (FGD 1, P2, Public EMS)

## Discussions

The purpose of this study was to explore and describe the experiences and challenges experienced by ECP’s in performing RSI within the pre-hospital setting. It became apparent that system-driven administrative demands and procedural complexity emerged as one of the prominent challenges to the effective execution of RSI in the pre-hospital setting. Participants frequently reported the mandatory requirement for clinical consultation with an on-call Doctor, particularly in private ambulance services, as a source of significant delays, frustration and professional dissatisfaction. While clinical oversight and governance structures are important for patient safety, the practical realities of obtaining authorisation in time-sensitive emergencies appear to undermine rather than enhance patient outcomes.

The participant accounts vividly illustrate how prolonged telephone consultations, sometimes lasting more than 30 min, can consume valuable time that would otherwise be dedicated to critical preparation and intervention. In the context of pre-hospital RSI, where timely airway management is directly linked to survival, such delays can increase the risk of hypoxia, aspiration and other adverse outcomes. Similar concerns have been reported in international literature, where excessive administrative requirements were found to delay life-saving interventions and reduce procedural success rates in the pre-hospital environment (Thomas et al. 2023). Moreover, mandatory consultations may inadvertently erode the professional autonomy of skilled ECPs, leaving them feeling undermined in front of patients and families, as echoed by participants in this study.

Beyond consultation requirements, participants also emphasised the importance of meticulous planning and preparation in minimising complications during RSI. Failure to systematically plan, prepare, check equipment, pre-oxygenate and mentally rehearse the procedure was identified as a critical factor that can contribute to adverse events, including hypoxic episodes (Lockey et al. 2017; Swinton et al. 2018). This aligns with existing evidence that preparation is a cornerstone of successful airway management; studies have consistently shown that inadequate pre-procedural planning increases the likelihood of complications such as multiple intubation attempts and peri-intubation hypoxia (Bernhard et al. 2015). In high-acuity environments, pre-planned checklists and structured preparation protocols have been demonstrated to improve technical performance, standardisation and patient safety (Burgess et al. 2018; Grant et al. 2021; Klingberg et al. 2020).

The role of unskilled assistance during RSI cannot be overstated as one of the prominent challenges to the effective execution of pre-hospital RSI. Participants reported that the lack of knowledgeable and experienced assistants frequently forced them to perform multiple critical tasks simultaneously, such as airway management, IV access and patient monitoring. This lack of coordinated team support significantly hindered their ability to focus on the technical aspects of RSI. Similar findings have been reported in international literature, where inadequate team training and skill discrepancies among crew members have been shown to compromise prehospital airway interventions (Trimmel et al. 2018). Studies emphasise that RSI is a complex, high-stakes intervention that requires well-rehearsed teamwork, with each team member understanding their role to ensure timely and effective execution (Crewdson et al. 2014; Crombie, George & McQueen 2015). When team members lack the necessary competence and skills, practitioners are often left to consume an unsustainable task burden, as reflected in the participant accounts.

Shortages of essential medications and equipment further compounded these challenges.

Several participants described encountering shortages of medications such as succinylcholine or reliance on malfunctioning basic equipment such as bag-valve masks. Medication shortages are a recurring challenge in low- and middle-income settings, with evidence showing that the unavailability of essential RSI medications often leads practitioners to improvise, potentially prolonging intubation attempts and exposing patients to increased hypoxia or hemodynamic instability (Bhattarai et al. 2023; Pillay & Hardcastle 2017; Sabogal & Tucker 2022).

The reliance on longer-acting neuromuscular blocking agents such as Rocuronium, as highlighted in the quotes, underscores the risks associated with medication limitations, especially during difficult intubations where rapid recovery is critical. Equipment failure in the pre-hospital setting has also been reported as a significant threat to airway management, with studies stressing the importance of functional and redundant airway devices to mitigate the risk of adverse outcomes (Crewdson et al. 2014; Prekker et al. 2014). These findings are consistent with the broader literature on task overload in emergency care, where the combination of inadequate staffing, lack of skilled assistance and equipment or medication constraints collectively strains the practitioner and increases cognitive load (Liu et al. 2024). In the pre-hospital context, where conditions are already unpredictable and resource limited, these factors increase the risk of procedural failure and poor patient outcomes.

Lastly, communication-related challenges and information gaps were identified as a significant challenge for ECPs performing RSI in the pre-hospital setting, with participants highlighting two main issues: lack of access to patient medical history and miscommunication among healthcare providers. Both factors were perceived as contributors to procedural difficulties and potential adverse outcomes. The absence of sufficient patient information, particularly medical history, was frequently reported as a barrier to safe and effective RSI. As several participants noted, patients encountered in the pre-hospital setting are often unconscious, unaccompanied or found in circumstances where bystanders are unable to provide reliable histories. In such cases, clinicians are forced to proceed without critical information, such as drug allergies, current medications or substance use, which increases the risk of inappropriate drug selection or adverse reactions.

Previous studies support this finding, demonstrating that limited availability of patient records in the pre-hospital environment can hinder decision-making, delay interventions and compromise patient safety (Bigham et al. 2012). The use of certain anaesthetic or paralytic drugs without knowledge of comorbidities or concurrent drug use has been shown to influence both drug efficacy and the likelihood of complications (Crewdson et al. 2014).

Miscommunication among healthcare providers further compounded the challenges of performing RSI.

Participants described instances where unclear instructions and lack of role clarity between crews led to confusion and errors during time-sensitive interventions. In high-acuity situations such as airway management, these breakdowns in communication can significantly increase the risk of failed intubation attempts, prolonged hypoxia or adverse hemodynamic events. This observation is consistent with evidence from the broader pre-hospital and emergency care literature, which highlights communication failures as a common contributor to adverse events and medical errors (Hohenstein et al. 2014; Wilk et al. 2018). Schmutz and Manser (2013) and Hohenstein et al. (2014) also emphasise that effective communication and teamwork are central to safe clinical practice, particularly during complex procedures that require coordinated effort across providers.

The findings from this study align with prior research indicating that structured communication strategies, such as pre-procedural briefings, closed-loop communication and standardised handover protocols, can mitigate the risks associated with miscommunication in emergency medical contexts (Starmer et al. 2014; Weller, Boyd & Cumin 2014).

### Limitations

This study was conducted in three selected provinces: Gauteng, KwaZulu-Natal and Western Cape covering ECPs from both public and private ambulance service providers. As such, the findings may not be representative of all ECP’s in South Africa, and results could differ if the study were conducted in other provinces.

## Conclusion

This study highlights how administrative requirements, task-overload and communication-related challenges collectively hinder the safe execution of pre-hospital RSI. Administrative demands, such as mandatory consultations and inadequate preparation time, delay time-critical interventions and compromise efficiency. Shortages of skilled assistance, limited equipment and high workload leave ECPs overextended and increase the risk of procedural errors. In addition, miscommunication among healthcare providers and the frequent lack of patient medical history further complicate care and elevate the risk of adverse outcomes. The insights gained can guide EMS leadership, stakeholders and policymakers in formulating targeted strategies such as optimising staffing models, ensuring consistent availability of essential resources, and strengthening operational support to enhance the safety, efficiency and quality of care delivered to critically ill or injured patients requiring RSI. Addressing these barriers is essential to support ECPs and enhance patient safety during pre-hospital RSI.

## References

[CIT0001] Beecham, G.B. & Kohn, M.D., 2025, *EMS airway management: Addressing challenges in adverse conditions*, StatPearls Publishing, viewed 06 April 2025, from https://www.ncbi.nlm.nih.gov/books/NBK557786/. 32491718

[CIT0002] Bernard, S.A., Nguyen, V., Cameron, P., Masci, K., Fitzgerald, M., Cooper, D. et al., 2010, ‘Prehospital rapid sequence intubation improves functional outcome for patients with severe traumatic brain injury: A randomized controlled trial’, *Annals of Surgery* 252(6), 959–965. 10.1097/SLA.0b013e3181efc15f 21107105

[CIT0003] Bernhard, M., Becker, T.K., Gries, A., Knapp, J. & Wenzel, V., 2015, ‘The first shot is often the best shot: First-pass intubation success in emergency airway management’, *Anesthesia & Analgesia* 118(4), 849–854. 10.1213/ANE.0000000000000891 26484464

[CIT0004] Bhattarai, H.K., Bhusal, S., Barone-Adesi, F. & Hubloue, I., 2023, ‘Prehospital emergency care in low- and middle-income countries: A systematic review’, *Prehospital and Disaster Medicine* 38(4), 495–512. 10.1017/S1049023X23006088 PMC10445116 37492946

[CIT0005] Bigham, B.L., Buick, J.E., Brooks, S.C., Morrison, M., Shojania, K.G. & Morrison, L.J., 2012, ‘Patient safety in emergency medical services: A systematic review of the literature’, *Prehospital Emergency Care* 17(4), 444–457. 10.3109/10903127.2011.621045 22128905

[CIT0006] Botha, J.C., Lourens, A. & Stassen, W., 2021, ‘Rapid sequence intubation: A survey of current practice in the South African prehospital setting’, *International Journal of Emergency Medicine* 14, 45. 10.1186/s12245-021-00368-3 PMC8369626 34404352

[CIT0007] Brown, C.A., Bair, A.E., Pallin, D.J. & Walls, R.M., 2015, ‘Techniques, success, and adverse events of emergency department adult intubations’, *Annals of Emergency Medicine* 65(4), 363–370. 10.1186/s13049-018-0554-6 25533140

[CIT0008] Burgess, Crewdson, K., Lockey, D.J. & Voelckel, W.G., 2018, ‘Pre-hospital anaesthesia: An updated survey of UK practice with emphasis on the role of standardisation and checklists’, *Emergency Medicine Journal* 31(7), 583–586. 10.1136/emermed-2017-206592 29794121

[CIT0009] Crewdson, K., Lockey, D.J., Voelckel, W., Temesvari, P. & Lossius, H.M., 2019, ‘Best practice advice on pre-hospital emergency anaesthesia and advanced airway management’, *Scandinavian Journal of Trauma, Resuscitation and Emergency Medicine* 27(1), 6. 10.1186/s13049-018-0554-6 PMC6341545 30665441

[CIT0010] Crombie, N., George, A. & McQueen, C., 2015, ‘Role allocation and team dynamics during pre-hospital rapid sequence induction of anaesthesia by a Physician- Critical Care Paramedic team in the United Kingdom: A 12-month review of practice’, *Journal of Anesthesia & Clinical Research* 6, 2. 10.4172/2155-6148.1000507

[CIT0011] Fevang, E., Perkins, Z., Lockey, D., Jeppesen, E. & Lossius, H.M., 2017, ‘A systematic review and meta-analysis comparing mortality in pre-hospital tracheal intubation to emergency department intubation in trauma patients’, *Critical Care* 21, 192. 10.1186/s13054-017-1787-x PMC5535283 28756778

[CIT0012] Grant, S., Pellatt, R.A., Shirran, M., Sweeny, A.L., Perez, S.R., Khan, G. et al., 2021, ‘Safety of rapid sequence intubation in an emergency training network’, *Emergency Medicine Australasia* 33(5), 857–867. 10.1111/1742-6723.13742 33565240

[CIT0013] Gunning, M., Perkins, Z., Crilly, J. & Rahden, R., 2013, ‘Paramedic rapid sequence induction (RSI) in a South African Emergency Medical Service: A retrospective observational study’, *South African Medical Journal* 103(9), 632–637. 10.7196/samj.6656 24300681

[CIT0014] Health Professions Council of South Africa (HPCSA), 2009, *Scope of practice: Emergency care practitioners*, Professional Board for Emergency Care, Pretoria, viewed 11 September 2025, from https://www.hpcsa.co.za/board/emergency-care.

[CIT0015] Hohenstein, C., Schultheis, K., Lotter, O., Fleischmann, T. & Hempler, D., 2014, ‘Communication and medication errors: Analysis of critical incident reports in prehospital emergency medicine (2005–2015)’, *Emergency Medicine Journal* 31(5), 415–418. 10.5847/wjem.j.1920-8642.2016.02.002 23349351

[CIT0016] Jarvis, J.L., Lyng, J.W., Miller, B.L., Perlmutter, M.C., Abraham, H. & Sahni, R., 2022, ‘Prehospital drug assisted airway management: An NAEMSP. Position statement and resource document’, *Prehospital Emergency Care* 26, 42–53. 10.1080/10903127.2021.1990447 35001829

[CIT0017] Jarvis, J.L., Panchal, A.R., Lyng, J.W., Bosson, N., Donofrio-Odmann, J.J., Braude, D.A., et al., 2024, ‘Evidence-based guideline for prehospital airway management, *Prehospital Emergency Care* 28(4), 545–557. 10.1080/10903127.2023.2281363 38133523

[CIT0018] Klingberg, C., Kornhall, D., Gryth, D., Kruger, A.J., Lossius, H.M. & Gellerfors, M., 2020, ‘Checklists in pre-hospital advanced airway management’, *Acta Anaesthesiologica Scandinavica* 64(1), 124–130. 10.1111/aas.13460 31436306

[CIT0019] Kovacs, G. & Sowers, N., 2018, ‘Airway management in trauma’, *Emergency Medicine Clinics of North America* 36(1), 61–84. 10.1016/j.emc.2017.08.006 29132582

[CIT0020] Liu, C-H., Yang, C-W., Lockey, A., Greif, R. & Cheng, A., 2024, ‘Factors influencing workload and stress during resuscitation – A scoping review’, *Resuscitation Plus* 18, 100630. 10.1016/j.resplu.2024.100630 PMC11711810 39790340

[CIT0021] Lockey, D.J., Crewdson, K., Davies, G., Jenkins, B., Klein, J., Laird, C., et al., 2017, ‘AAGBI: Safer pre-hospital anaesthesia 2017’, *Anaesthesia* 72(3), 379–390. 10.1111/anae.13779 PMC5324693 28045209

[CIT0022] National Department of Health, 2015, *Regulations relating to standards for emergency medical services*, Government Gazette, Pretoria, viewed 30 July 2025, from https://www.gov.za/sites/default/files/gcis_document/201712/41287gon1320.pdf.

[CIT0023] Okubo, M., Gibo, K., Hagiwara, Y., Nakayama, Y. & Hasegawa, K., 2017, ‘The effectiveness of rapid sequence intubation (RSI) versus non-RSI in emergency department: An analysis of multicenter prospective observational study’, *International Journal of Emergency Medicine* 10(1), 1–9. 10.1186/s12245-017-0129-8 PMC5267589 28124199

[CIT0024] Olvera, D.J., Stuhlmiller, D.F.E., Wolfe, A., Swearingen, C.F., Pennington, T. & Davis, D.P., 2018, ‘A continuous quality improvement airway program results in sustained increases in intubation success’, *Prehospital Emergency Care* 22(5), 602–607. 10.1080/10903127.2018.1433734 29465279

[CIT0025] Pillay, L. & Hardcastle, T., 2017, ‘Collective review of the status of rapid sequence intubation drugs of choice in trauma in low- and middle-income settings (prehospital, emergency department, and operating room setting)’, *World Journal of Surgery* 41, 1184–1192. 10.1007/s00268-016-3712-x 27646281

[CIT0026] Prekker, E.M., Kwok, H., Shin, J., Carlbom, D., Grabinsky, A. & Rea, D.T., 2014, ‘The process of prehospital airway management: Challenges and solutions during paramedic endotracheal intubation’, *Annals of Emergency Medicine* 58(6), 612–620. 10.1097/CCM.0000000000000213 PMC4902016 24589641

[CIT0027] Republic of South Africa. Department of Health, 2003, *The National Health Act of South Africa, 61 of 2003 Section 47 of the National Health Act*, Department of Health, Pretoria, viewed 09 July 2025, from https://www.gov.za/sites/default/files/gcis_document/201409/a61-03.pdf.

[CIT0028] Sabogal de la Pava, M.L. & Tucker, E.L., 2022, ‘Drug shortages in low- and middle-income countries: Colombia as a case study’, *Journal of Pharmaceutical Policy and Practice* 15, 42. 10.1186/s40545-022-00439-7 PMC9189796 35698240

[CIT0029] Schmutz, J. & Manser, T., 2013, ‚Do team processes really have an effect on clinical performance? A systematic literature review’, *British Journal of Anaesthesia* 110(4), 529–544. 10.1093/bja/aes513 23454826

[CIT0030] Starmer, A.J., Spector, N.D., Srivastava, R., Allen, A.D., Landrigan, C.P. & Sectish, T.C., 2014, ‘Changes in medical errors after implementation of a handoff program’, *New England Journal of Medicine* 371(19), 1803–1812. 10.1056/NEJMsa1405556 25372088

[CIT0031] Stein, C., Botha, M., Kramer, E., Nevin, D., Dickerson, R., Goldstein, L. et al., 2011, ‘Position statement prehospital rapid sequence intubation’, *South African Medical Journal* 101(3), 163. 10.7196/SAMJ.4230 21382243

[CIT0032] Swinton, P., Corfield, A.R., Moultrie, C., Percival, D., Proctor, J., Sinclair, N. et al., 2018, ‘Impact of drug and equipment preparation on pre-hospital emergency anaesthesia procedural time, error rate and cognitive load’, *Scandanavian Journal of Trauma, Resuscitation and Emergency Medicine* 26, 82. 10.1186/s13049-018-0549-3 PMC6150998 30241559

[CIT0033] Thomas, B., Urban, S., Banker, J., Shah, A., Schaid, T., Wright, A. et al., 2023, ‘Tick-tock: Prehospital intubation is associated with longer field time without any survival benefit’, *Trauma/Critical Care* 174(4), 1034–1040. 10.1016/j.surg.2023.06.021 37500409

[CIT0034] Trimmel, H., Beywinkler, C., Hornung, S., Kreutziger, J. & Voelckel, W.G., 2018, ‘Success rates of pre-hospital difficult airway management: A quality control study evaluating an in-hospital training program’, *International Journal of Emergency Medicine* 11(1), 19. 10.1186/s12245-018-0178-7 PMC5856681 29549460

[CIT0035] Wallace, C. & McGuire, B., 2014, ‘Rapid sequence induction: Its place in modern anaesthesia’, *Continuing Education in Anaesthesia Critical Care & Pain* 14(3), 130–135. 10.1093/bjaceaccp/mkt047

[CIT0036] Weller, J., Boyd, M. & Cumin, D., 2014, ‘Teams, tribes, and patient safety: Overcoming barriers to effective teamwork in healthcare’, *Postgraduate Medical Journal* 90(1061), 149–154. 10.1136/postgradmedj-2012-131168 24398594

[CIT0037] Wilk, S., Siege, L., Siege, K. & Hohenstein, C., 2018, ‘Miscommunication as a risk focus in patient safety: Work process analysis in prehospital emergency care’, *Der Anaesthesist* 67(4), 255–263. 10.1007/s00101-018-0413-x 29404658

